# Sex-Differences in the Metabolic Health of Offspring of Parents with Diabetes: A Record-Linkage Study

**DOI:** 10.1371/journal.pone.0134883

**Published:** 2015-08-26

**Authors:** Marian C. Aldhous, Rebecca M. Reynolds, Archie Campbell, Pamela Linksted, Robert S. Lindsay, Blair H. Smith, Jonathan R. Seckl, David J. Porteous, Jane E. Norman, Generation Scotland

**Affiliations:** 1 Tommy’s Centre for Maternal and Fetal Health, MRC Centre for Reproductive Health, Queen’s Medical Research Institute, University of Edinburgh, Edinburgh, United Kingdom; 2 Endocrinology Unit, University/BHF Centre for Cardiovascular Science, Queen’s Medical Research Institute, University of Edinburgh, Edinburgh, United Kingdom; 3 Generation Scotland, Centre for Genomic and Experimental Medicine, Institute of Genetics & Molecular Medicine, University of Edinburgh, Western General Hospital, Edinburgh, United Kingdom; 4 British Heart Foundation Glasgow Cardiovascular Research Centre, University of Glasgow, Glasgow, United Kingdom; 5 Medical Research Institute, University of Dundee, Dundee, United Kingdom; 6 Medical Genetics Section, Centre for Genomic and Experimental Medicine, Institute of Genetics and Molecular Medicine, University of Edinburgh, Western General Hospital, Edinburgh, United Kingdom; 7 Generation Scotland: A Collaboration between the University Medical Schools and NHS in Aberdeen, Dundee, Edinburgh and Glasgow, Scotland, United Kingdom; Hunter College, UNITED STATES

## Abstract

Maternal diabetes in pregnancy affects offspring health. The impact of parental diabetes on offspring health is unclear. We investigated the impact of parental diabetes on the metabolic-health of adult-offspring who did not themselves have diabetes. Data from the Generation Scotland: Scottish Family Health Study, a population-based family cohort, were record-linked to subjects’ own diabetes medical records. From F_0_-parents, we identified F_1_-offspring of: mothers with diabetes (OMD, n = 409), fathers with diabetes (OFD, n = 468), no parent with diabetes (ONoPD, n = 2489). Metabolic syndrome, body, biochemical measurements and blood-pressures were compared between F_1_-offspring groups by sex. A higher proportion of female OMD had metabolic syndrome than female OFD or ONoPD (P<0.0001). In female offspring, predictors of metabolic syndrome were: having a mother with diabetes (OR = 1.78, CI 1.03–3.07, [reference ONoPD]), body mass index (BMI, OR = 1.21, CI 1.13–1.30) and age (OR = 1.03, CI 1.01–1.06). In male offspring, predictors of metabolic syndrome were: BMI (OR = 1.18, CI 1.09–1.29) and percent body-fat (OR = 1.12, CI 1.05–1.19). In both sexes, OMD had higher blood-pressures than OFD (P<0.0001). In females, OMD had higher glucose (P<0.0001) and percent body-fat (P<0.0001) compared with OFD or ONoPD. OMD and OFD both had increased waist-measurements (P<0.0001), BMI (P<0.0001) and percent body-fat (P<0.0001) compared with ONoPD. Female OMD and OFD had lower HDL-cholesterol levels (P<0.0001) than female ONoPD. Parental diabetes is associated with higher offspring-BMI and body-fat. In female offspring, maternal diabetes increased the odds of metabolic syndrome, even after adjusting for BMI. Further investigations are required to determine the mechanisms involved.

## Introduction

Population-rates of diabetes and obesity have risen in recent years [1]. Obesity increases the risk of type-2 diabetes mellitus (T2DM); having a parent with diabetes increases the risks of later obesity, T2DM and/or cardiovascular problems in their children [2–4]. Maternal diabetes during pregnancy, whether pre-existing type-1 diabetes mellitus (T1DM), T2DM or gestational diabetes mellitus (GDM, first diagnosed during pregnancy) has long-term health-effects on the offspring. Such offspring have higher birth-weights [5–7] and increased adiposity during childhood compared with offspring of mothers without diabetes [8,9]. In contrast, children of fathers with diabetes have lower birth-weights [10,11]. In Scotland, ~5% of women of reproductive age have been diagnosed with diabetes [12], which may impact childhood obesity-rates and health. As childhood obesity-rates are already high (16.8% in Scotland in 2012 [13]) this raises public-health concerns.

The longer-term health-effects of parental diabetes on adult offspring are less clear. Increased body mass index (BMI) was seen in 18 year-old boys exposed to intrauterine maternal diabetes compared with their older brothers, who were born before their mothers developed diabetes [14]. However, there are few studies of long-term effects of maternal diabetes diagnosed after pregnancy or paternal diabetes on adult offspring and few data comparing the impact of paternal with maternal diabetes.

The Generation Scotland: Scottish Family Health Study (GS:SFHS) is a family-based population cohort, recruited to investigate the genetic and environmental factors involved in the aetiology of various complex diseases common in Scotland [15,16]. The known extended family-relationships within GS:SFHS also allow for investigations of any trans-generational effects of grandparental T2DM. Having a grandparent with diabetes has been associated with children being overweight aged 4 years [17], but there is little information on any long-term effects into adulthood.

The primary aim of this study was to determine, in adults without diabetes, the long-term metabolic health-effects of having a mother with diabetes compared with having a father with diabetes and no parent with diabetes. Specifically, we determined the frequency of metabolic syndrome (MetS) and the relative contributions of body and biochemical measurements, blood-pressure and social factors. The secondary aims were to investigate whether intrauterine exposure to maternal diabetes had any additional effect on offspring health compared with having a mother with diabetes diagnosed after pregnancy, and to investigate whether having a grandparent with diabetes affected body, biochemical measurements, or blood-pressure of their grandchildren through specific maternal- or paternal-lineages.

## Methods

### Generation Scotland: Scottish Family Health Study Cohort

GS:SFHS participants were recruited from the Scottish general population during 2006–2011, via primary-care physicians and publicity across Scotland, resulting in 23,960 people in family groups. Potential study participants were screened to exclude those with serious terminal illness or inability to give informed consent (~5% excluded). At recruitment into GS:SFHS, participants gave written consent, completed a detailed health and social questionnaire and attended a research clinic for clinical examination, body measurements and collection of fasting blood samples. Details of the GS:SFHS cohort and the data-collection processes are published elsewhere [15,16] and via links from the “GS Resources” page of the Generation Scotland website (www.generationscotland.org). GS data are available for medical research on application to the GS Access Committee. Details of access to GS data are available on the “Access” page of the Generation Scotland website. This was a data-linkage analysis, so no new data were collected for this study.

### Approvals

For this study, only those GS:SFHS participants who had given written consent for record-linkage of their GS:SFHS data to their own medical records were used. Ethical approval for the record-linkage was obtained from the East of Scotland Research Ethics Committee (reference 10/S1402/20). Permissions for use of NHS health data in this record-linkage were obtained from the NHS Privacy Advisory Committee, Scottish Diabetes Research Network and Caldicott Guardians for Scotland.

### Record linkage process

**Fig 1:**
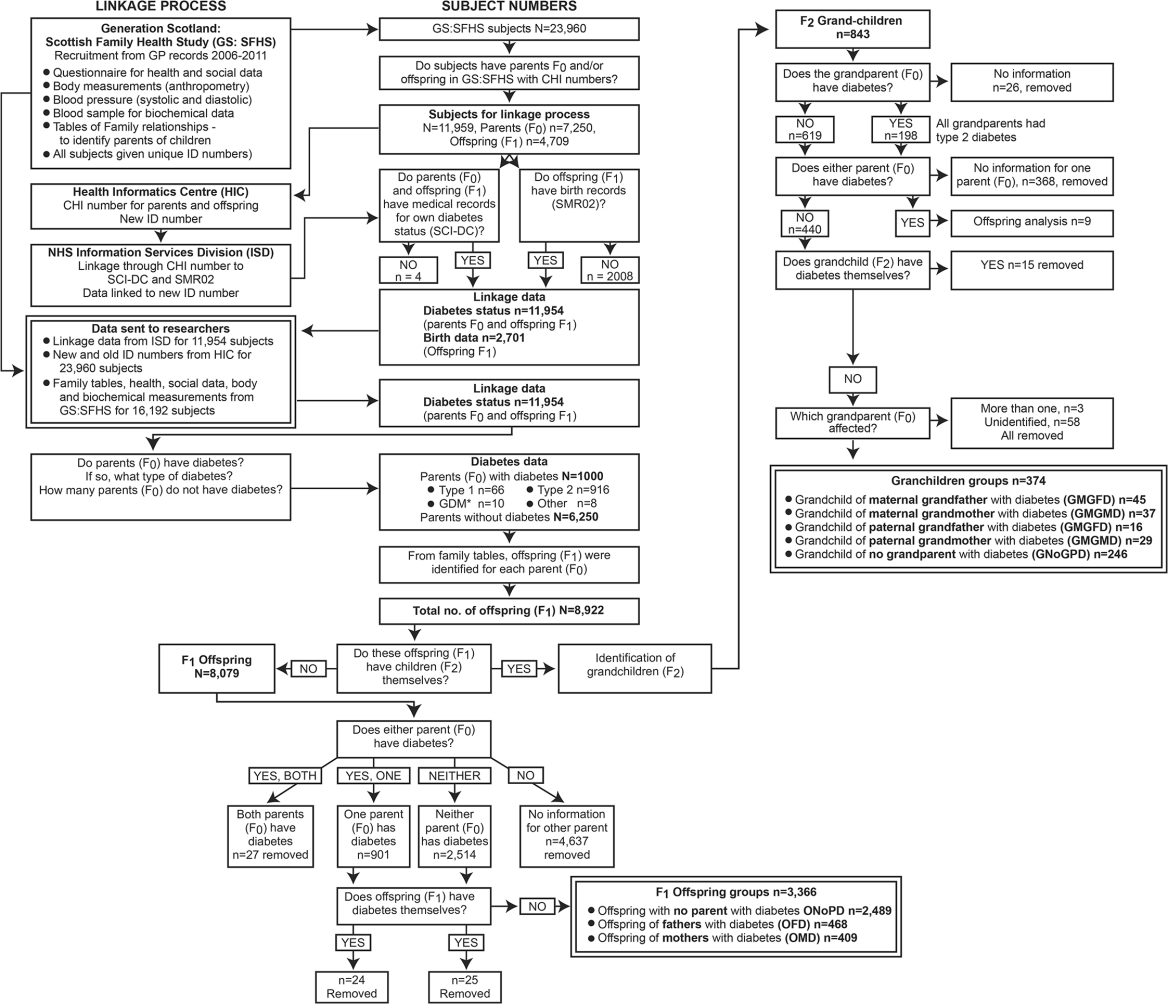
Summary of the linkage process and identification of the F_1_-offspring and F_2_-grandchildren groups for analysis. Linkage process of GS:SFHS data. Subjects who had children or parents within the GS:SFHS cohort and had health numbers were sent via HIC to ISD for linkage to their own diabetes and birth medical records. From the diabetes-linked data, the F_1_-offspring of the F_0_-parents with or without diabetes were identified. F_1_-offspring who had children of their own were separated for analysis of the effects of grandparental diabetes on F_2_-grandchildren. F_1_-offspring groups were defined by whether their mother (OMD) or father (OFD) or no parent had diabetes (ONoPD). F_2_-grandchildren groups with grandparents with diabetes were grouped by maternal or paternal lineage: grandchild of maternal grandmother with diabetes (GMGMD), grandchild of maternal grandfather with diabetes (GMGFD), grandchild of paternal grandmother with diabetes (GPGMD), grandchild of paternal grandfather with diabetes (GPGFD), or grandchild of grandparents with no diabetes (GNoGPD). * Mothers who developed GDM during pregnancy were identified from SMR02

Data were linked to records for diabetes diagnoses at any time-point to November 2011 (via the Scottish Care Information Diabetes Collaboration [SCI-DC, http://www.sci-diabetes.scot.nhs.uk/]) and pregnancy/birth records (Scottish Morbidity Report 02 [SMR02] from Information Services Division [ISD], NHS Scotland). To safeguard subject confidentiality, data were linked through the Health Informatics Centre (HIC) Dundee and ISD. The record-linkage process is summarized in Fig 1, showing identification of subjects at each stage and final numbers for each subject group. GS:SFHS ID-numbers for participants with F_0_-parents and/or F_1_-offspring within GS:SFHS were sent to HIC, where participant NHS-health numbers were identified, assigned new ID-numbers and sent to ISD for record-linkage. GS:SFHS pedigree tables were used to identify F_0_-parents, F_1_-offspring and F_2_-grandchildren.

### GS:SFHS data analysed

Data were obtained at recruitment into GS:SFHS [16], from direct measurement (body, biochemistry, and blood-pressure) or questionnaire (comorbidity information for self and family members, family relationships, smoking status and postcode at recruitment). Over 95% of participants were Caucasian. Parameters analysed were:

#### Body measurements

height, weight, BMI, waist, hips, waist: hip ratio (WHR), percent body-fat, systolic and diastolic blood-pressure (both mean of two measurements).

#### Blood biochemical measurements

sodium, potassium, urea, creatinine, glucose, total cholesterol, HDL-cholesterol, total: HDL cholesterol ratio (THCR).

#### Diseases diagnosed

diabetes, asthma, depression, *cardiovascular disease* (heart disease, hypertension, stroke); *bone disease* (osteoarthritis, rheumatoid arthritis, hip fracture).

#### Smoking data

smoking status at recruitment (current, non-smokers, ex-smokers); exposure to other peoples’ smoke.

#### Scottish Index of Material Deprivation (SIMD)

categorized in quintiles (1 [most deprived] to 5 [least deprived], from 2009) for each subject’s home postcode at recruitment (http://www.scotland.gov.uk/Topics/Statistics/SIMD).

### Identification of F_1_-offspring and F_2_-grandhcildren of F_0_-parents with diabetes

From SMR02 data, we identified F_1_-offspring whose F_0_-mothers developed GDM during the index pregnancy (n = 10) and so were exposed *in utero* (EIU) to maternal diabetes. From SCI-DC data, we obtained diabetes diagnoses (with type and dates) for all subjects in the record-linkage, i.e. a diagnosis (or no diagnosis) of diabetes in the subjects’ own medical records. Offspring whose mothers had pre-existing T1DM (n = 8) during the index pregnancy were also included in the EIU group. No mothers had developed T2DM prior to the index pregnancy. Due to small numbers, the EIU group was combined with those whose mothers developed diabetes after pregnancy as F_1_-offspring of mothers with diabetes (OMD) for all analyses: OMD were compared with F_1_-offspring of fathers with diabetes (OFD) and F_1_-offspring of no parent with diabetes (ONoPD).

The GS:SFHS questionnaire asked whether the subject or family members had diabetes: F_1_-offspring (n = 49) or F_2_-grandchildren (n = 5) who had diabetes themselves were removed because we aimed to investigate the effect of familial diabetes on the subjects’ health, which might be obscured by subjects’ own diabetes disease-processes. F_1_-offspring with both F_0_-parents with diabetes (n = 27) and F_2_-grandchildren with more than one F_0_-grandparent with diabetes (n = 3) were removed. Neither F_1_-parent of the F_2_-grandchildren had diabetes. Siblings were included but known twins were removed.

F_1_- offspring were grouped according to parental diabetes status:

F_1_-offspring of mothers with diabetes: (OMD, n = 409) those whose mothers were diagnosed with diabetes;
F_1_-offspring of fathers with diabetes: (OFD, n = 468) those whose fathers were diagnosed with diabetes;
F_1_-offspring of no parent with diabetes: (ONoPD, n = 2,489) those for whom neither parent had diabetes.

F_2_-grandchildren were grouped according to parental lineage:

F_2_-Grandchildren of maternal grandfather with diabetes: (GMGFD, n = 45)
F_2_-Grandchildren of maternal grandmother with diabetes: (GMGMD, n = 37)
F_2_-Grandchildren of paternal grandfather with diabetes: (GPGFD, n = 16)
F_2_-Grandchildren of paternal grandmother with diabetes: (GPGMD, n = 29)
F_2_-Grandchildren of grandparents with no evidence of diabetes: (GNoGPD, n = 246).

### Definition of Metabolic Syndrome (MetS)

The International Diabetes Federation definitions (from 2009 [18]) were used to define those with MetS. Using these criteria, subjects were defined as having MetS if three of five criteria are present:

- Central obesity: a waist measurement of ≥94cm in males and ≥80cm in females;
- Reduced HDL-cholesterol: ≤1.0 mmol/L (40mg/dL) in males and ≤1.3 mmol/L (50mg/dL) in females;
- Elevated blood-pressure: systolic ≥130 and/or diastolic ≥85 mm Hg and/or being treated with anti-hypertensive drugs;
- Raised glucose levels: ≥5.6 mmol/L (100mg/dL);
- Elevated triglyceride levels: >1.7mmol/L (150mg/dL).

Triglyceride levels were not available for our subjects: hence if subjects met only two (of four) criteria they were assumed not to have MetS. Where values for any other criteria were missing, subjects were only included if the three remaining criteria were all outside the thresholds for MetS (i.e. had MetS) or were all within normal values (i.e. did not have MetS).

### Statistical analysis

To investigate the relationships of different parameters in the F_1_-offspring or F_2_-grandchildren groups, Kruskal-Wallis tests were used to analyse continuous measurements. Chi-squared tests were used for categorical data and Spearman tests were used for correlations; statistical significance was set at P<0.05. Given the major effect of sex of the subjects on the outcomes of interest, all analyses were performed subdivided by sex of offspring or grandchildren. Rest of cohort (RoC, GS:SFHS subjects without diabetes excluded from the study groups) data were included in the graphs to show the representativeness of the offspring data. MetS was compared between the F_1_-offspring groups by sex.

To determine the predictors of MetS, relevant body and biochemical measurements, F_1_-offspring group (ONoPD as reference), age (continuous variable), smoking, SIMD (quintile 5 as reference), whether a sibling was in the study (yes/no) were used in forwards and backwards stepwise logistic regression (alpha of 0.5) to identify factors associated with MetS. Statistical analyses used GraphPad Prism6 (GraphPad Software) and Minitab 16 Statistical Software (Minitab Inc.).

## Results

### Record-linkage

Diabetes-linked data were obtained for 11,954 GS;SFHS subjects. SMR02 provided data for F_1_-offspring and F_2_-grandchildren (born 1980–1992) for whether their mother had GDM, their mother’s age at pregnancy, mother’s height, SIMD quintile at birth, birth-weight (not adjusted for gestational age), year and month of birth (for season of birth); data on their mother’s smoking history were incomplete and not used; the mother’s weight or BMI at pregnancy were not recorded in SMR02 at that time.

### Demographics, comorbidity, social and birth data

Demographic, comorbidity, social and birth data were compared by sex between F_1_-offspring groups (Table 1). The median age of all the F_1_-offspring was 33 years (interquartile range [IQR] 26–40 years). OMD and OFD were significantly older than ONoPD (Kruskal-Wallis, P<0.0001 for both sexes). There were no differences between F_1_-offspring groups in the proportion of subjects who had siblings in the study for either sex.

**Table 1:** Demographics, comorbidity, social/lifestyle and birth data for male and female F_1_-offspring.

	Males in F_1_-Offspring groups			Χ^2^	P value	Females in F_1_-Offspring groups			Χ^2^	P value
	OMD	OFD	ONoPD			OMD	OFD	ONoPD		
Demographic data	N = 164	N = 194	N = 1078			N = 245	N = 274	N = 1411		
*Sex (% group)*	40.1	41.5	43.3			59.9	58.5	56.7	1.807	0.4051 ^a^
*Age (years)*:										
Median	40.0	35.0	31.0		**<0.0001** ^**b**^	39.0	36.0	32.0		**<0.0001** ^**b**^
IQR ^**c**^	31.3–48.0	28.0–43.0	25.0–38.0			31.5–46.0	28.0–42.0	25.0–39.0		
Range	18–61	18–61	18–60			18–65	18–59	18–62		
No. (%) aged 18–29	32 (19.5)	54 (27.8)	446 (41.4)			50 (20.4)	81 (29.6)	561 (39.8)		
No. (%) aged 30–40	51 (31.1)	81 (41.8)	442 (41.0)	**95.06**	**<0.0001**	84 (34.3)	110 (40.1)	587 (41.6)	**96.69**	**<0.0001**
No. (%) aged >41	81 (49.4)	59 (30.4)	190 (17.6)			111 (45.3)	83 (30.3)	263 (18.6)		
*Sibling in study n (%)*										
Yes	115 (70.1)	140 (72.3)	820 (76.1)	3.467	0.1767	189 (77.1)	207 (75.5)	1104 (78.2)	1.016	0.6016
No	49 (29.9)	54 (27.7)	258 (23.9)			56 (22.9)	67 (24.5)	307 (21.8)		
**Comorbidity data**										
No. (%) with data	161 (98.2)	180 (92.8)	1048 (97.2)			239 (97.6)	269 (98.2)	1391 (98.6)		
*Other diseases n (%)* **:**				**48.09** ^**d**^	**<0.0001**				**53.12** ^**d**^	**<0.0001**
Asthma	19 (11.2)	26 (14.1)	158 (14.8)	0.3569 ^**e**^	0.8366	55 (12.8)	64 (13.7)	363 (14.4)	1.045 ^**e**^	0.5929
Cardiovascular disease ^f^	21 (12.4)	6 (3.3)	25 (2.3)	**41.25** ^**e**^	**<0.0001**	43 (9.8)	25 (5.3)	73 (2.9)	**20.62** ^**e**^	**<0.0001**
Depression	11 (6.5)	15 (8.2)	59 (5.5)	2.626 ^**e**^	0.2691	45 (10.4)	42 (9.0)	183 (7.3)	**8.614** ^**e**^	**0.0135**
Bone disease ^g^	6 (3.6)	8 (4.3)	26 (2.4)	3.115 ^**e**^	0.2106	23 (5.3)	17 (3.6)	46 (1.8)	**33.35** ^**e**^	**<0.0001**
No other disease	112 (66.3)	129 (70.1)	801 (74.8)			261 (60.6)	319 (68.2)	1842 (73.2)		
**Social/lifestyle data**										
*Smoking status*										
No. (%) with data	157 (96.6)	178 (91.8)	1041 (96.6)			237 (96.7)	266 (97.1)	1379 (97.7)		
Current n (%)	42 (26.7)	47 (26.4)	197 (18.9)			40 (16.9)	49 (18.4)	182 (13.2)		
Never n (%)	75 (47.8)	75 (42.1)	528 (50.7)	**10.72**	**0.0298**	122 (51.5)	142 (53.4)	691 (50.1)	**11.30**	**0.0234**
Ex n (%)	40 (25.5)	56 (31.5)	316 (30.4)			75 (31.6)	75 (28.2)	506 (36.7)		
*Exposure to other peoples’ smoke*										
No. (%) with data	152 (92.7)	167 (86.1)	1002 (92.9)			219 (89.4)	245 (89.4)	1289 (91.4)		
Yes	92 (60.5)	78 (46.7)	495 (49.4)	**7.544**	**0.0230**	112 (51.1)	101 (41.2)	539 (41.8)	**6.972**	**0.0306**
No	60 (39.5)	89 (53.3)	507 (50.6)			107 (48.9)	144 (58.8)	750 (58.2)		
*SIMD quintile at recruitment*										
No. (%) with data	147 (89.6)	171 (88.1)	960 (89.1)			212 (86.5)	244 (89.1)	1264 (89.6)		
1^st^ (Most deprived)	26 (17.7)	28 (16.4)	71 (7.4)			37 (17.4)	32 (13.1)	113 (8.9)		
2^nd^	23 (15.6)	20 (11.7)	128 (13.3)			40 (18.9)	44 (18.0)	180 (14.2)		
3^rd^	18 (12.2)	34 (19.9)	165 (17.2)	**30.88**	**0.0001**	48 (22.6)	33 (13.5)	198 (15.7)	**37.95**	**<0.0001**
4^th^	37 (25.2)	34 (19.9)	266 (27.7)			43 (20.3)	65 (26.6)	363 (28.7)		
5^th^ (Least deprived)	43 (29.3)	55 (32.1)	330 (34.4)			44 (20.8)	70 (28.7)	410 (32.4)		
**Birth (SMR02) data**										
*Maternal height (cm)*										
No. (%) with data	25 (15.2)	34 (17.5)	327 (30.3)			38 (15.9)	61 (21.6)	433 (31.6)		
Median	163.0	162.0	163.0		0.1900 ^**b**^	160.0	160.0	163.0		**<0.0001** ^**b**^
IQR	157.5–165.0	157–166.3	158.0–166.0			155.8–163.0	156.0–165.0	159.0–166.0		
*Baby SIMD quintile at birth*										
No (%). with data	25 (15.2)	37 (19.1)	336 (31.2)			36 (14.7)	61 (22.3)	431 (30.5)		
1^st^ (Most deprived)	6 (24.0)	5 (13.5)	33 (9.8)			10 (27.8)	13 (21.3)	41 (9.5)		
2^nd^	3 (12.0)	9 (24.3)	41 (12.2)			6 (16.7)	10 (16.4)	69 (16.0)		
3^rd^	5 (20.0)	3 (8.1)	50 (14.9)	11.88	0.1565	5 (13.9)	6 (9.8)	82 (19.0)	**12.86**	**0.0109**
4^th^	4 (16.0)	10 (27.0)	97 (28.9)			8 (22.2)	18 (29.5)	106 (24.6)		
5^th^ (Least deprived)	7 (28.0)	10 (27.0)	115 (34.2)			7 (19.4)	14 (23.0)	133 (30.9)		
**Parental Data**										
*Parental Diabetes Type*										
Type 1	7 (4.3) ^h^	3 (1.5)				12 (4.9) ^h^	9 (3.3)			
Type 2	149 (90.8)	190 (97.9)	NA	**10.45**	**0.0151**	227 (92.7)	267 (96.3)	NA	5.948	0.1142
GDM	6 (3.7) ^j^	0				4 (1.6)	0			
Unknown type	2 (1.2)	1 (0.5)				2 (0.8)	1 (0.4)			
*Decade of parental diabetes’ diagnosis*										
1950–1979	2 (1.2)	1 (0.5)				6 (2.4)	2 (0.7)			
1980–1999	33 (20.1)	40 (20.6)	NA	1.383	0.7096	51 (20.8)	68 (24.8)	NA	3.732	0.2920
2000–2011	129 (78.7)	152 (78.4)				187 (76.3)	202 (73.7)			
Date unknown	0	1 (0.5)				1 (0.4)	2 (0.7)			
*Maternal BMI*										
No. (%) with data	140 (85.4)	108 (55.7)	1015 (94.2)			204 (83.2)	177 (64.6)	1318 (93.4)		
Median	30.5	27.1	25.4		**<0.0001** ^**b**^	30.5	28.0	26.0		**<0.0001** ^**b**^
IQR	26.8–35.2	23.6–31.1	23.0–29.0			26.1–35.1	24.6–32.2	23.2–29.3		
No. (%) BMI <25	21 (15.0)	38 (35.2)	453 (44.6)			42 (20.6)	52 (29.4)	543 (41.2)		
No. (%) BMI 25–29	43 (30.7)	37 (34.3)	357 (35.2)	**87.24**	**<0.0001**	50 (24.5)	60 (33.9)	496 (37.6)	**114.3**	**<0.0001**
No. (%) BMI >30	76 (54.3)	33 (30.5)	205 (20.2)			112 (54.9)	65 (36.7)	279 (21.2)		
*Paternal BMI*										
No. (%) with data	50 (30.5)	173 (89.2)	1033 (95.8)			78 (31.8)	247 (90.1)	1325 (93.9)		
Median	27.3	29.4	26.7		**<0.0001** ^**b**^	25.9	30.9	26.9		**<0.0001** ^**b**^
IQR	25.1–32.5	26.7–33.3	24.7–29.0			24.1–31.3	27.6–34.4	24.6–29.2		
No. (%) BMI <25	12 (24.0)	17 (9.8)	285 (27.6)			26 (33.3)	19 (7.7)	373 (28.2)		
No (%) BMI 25–29	18 (36.0)	80 (46.2)	557 (53.9)	**71.68**	**<0.0001**	26 (33.3)	91 (36.8)	677 (51.1)	**144.9**	**<0.0001**
No. (%) BMI >30	20 (40.0)	76 (43.9)	191 (18.5)			26 (33.3)	137 (55.5)	275 (20.7)		

^**a**^ Chi-square and p value given from comparison with females

^**b**^ Kruskal-Wallis analysis

^c^ IQR denotes interquartile range

^**d**^ All ‘other diseases’ together

^**e**^ χ^2^ and p values from each disease individually (vs. no other disease)

^**f**^ Cardiovascular disease included heart disease, hypertension and stroke

^**g**^ Bone disease included history of hip fracture, osteoarthritis and rheumatoid arthritis

^h^ Includes those whose mothers developed type 1 diabetes before the offspring was born (n = 8); these offspring were exposed *in utero* (EIU) to diabetes.

^**j**^ Two mothers with GDM developed T2D at a later date

NA denotes not applicable

#### Comorbidity

Differences in proportions of subjects with other diseases were seen between the F_1_-offspring groups in both males and females (P<0.0001). Higher percentages of OMD had cardiovascular disease than OFD and ONoPD (P<0.0001 for both sexes). In females, but not males, higher percentages of OMD and OFD than ONoPD had depression (P = 0.0135) or bone disease (P<0.0001).

#### Smoking

Smoking status differed between the groups in males (P = 0.0298) and females (P = 0.0234), as higher percentages of OMD and OFD were current smokers than ONoPD. A higher proportion of OMD than OFD or ONoPD were exposed to other peoples’ smoke in males (P = 0.0230) and females (P = 0.0306).

#### SIMD quintile at recruitment

Material deprivation differed between groups (P<0.0001 for both sexes). In males and females, higher percentages of OMD and OFD than ONoPD lived in the most deprived areas.

#### SMR02 (birth) data

In females, maternal height at pregnancy was lower in OMD and OFD compared with ONoPD (Kruskal-Wallis P<0.0001). Higher percentages of female, but not male, OMD and OFD than ONoPD were born into the most deprived areas (P = 0.0109). There were no significant differences in the mother’s age at pregnancy, birth-weight or season of birth between the groups.

### Parental data

#### Parental diabetes type

The numbers of F_1_-offspring whose fathers (n = 12) or mothers (n = 19) had T1DM were too small to compare with F_1_-offspring whose fathers or mothers had T2DM (Table 1). All diabetes types were combined within the OMD and OFD groups. There was an increase in the numbers of parents diagnosed with diabetes across the decades: parents diagnosed with diabetes before 1979 all had T1DM; for parents diagnosed in 1980–1999, most had T2DM and a few had T1DM (n = 15), whereas the majority of the parents with diabetes (~75%) were diagnosed with T2DM after 2000.

#### Maternal BMI

Maternal BMI was higher in OMD than the other two groups (Kruskal-Wallis P<0.0001) for both sexes. A higher proportion of OMD had mothers who were overweight (BMI of 25–29) or obese (BMI ≥30) than OFD and ONoPD (85% vs. 64.8% and 55.4% respectively in males; 79.4% vs. 70.6% and 58.8% respectively in females).

#### Paternal BMI

Paternal BMI was higher in OFD than the other two groups (Kruskal-Wallis P<0.0001) for both sexes. A higher proportion of OFD had fathers who were overweight or obese than OMD and ONoPD (90.2% vs. 76.0% and 72.4% respectively in males; 92.3% vs. 66.6% and 71.8% respectively in females).

### Metabolic syndrome

Higher percentages of female OMD or female OFD had MetS than female ONoPD (Table 2). In male offspring, differences did not quite reach statistical significance. Comparison of OFD with ONoPD showed higher percentages of OFD with MetS in male (P = 0.0358) and female (P = 0.0285) offspring.

**Table 2:** Results of univariate and multivariate analysis of metabolic syndrome in the F_1_-offspring groups, analysed by sex.

Univariate analysis	Males					Females				
	**No. of criteria met for**		**Total**	**Χ** ^**2**^	**P value**	**No. of criteria met for**		**Total**	**Χ** ^**2**^	**P value**
***F*_*1*_*-Offspring group***	**Metabolic syndrome**		**(N)**			**Metabolic syndrome**		**(N)**		
	**0–2**	**≥3**				**0–2**	**≥3**			
**OMD n (%)**	123 (88.5)	16 (11.5)	139			162 (79.4)	42 (20.6)	204		
**OFD n (%**	141 (87.6)	20 (12.4)	161	5.911	0.0521	204 (88.7)	26 (11.3)	230	**38.42**	**<0.0001**
**ONoPD n (%)**	840 (92.5)	68 (7.5)	908	**2.099** ^**a**^	**0.0358** ^**a**^	1090 (92.9)	83 (7.1)	1173	**4.798** ^**a**^	**0.0285 ^a^**
**Total N (%)**	1104 (91.4)	104 (8.6)	1208			1456 (90.6)	151 (9.4)	1607		
**Logistic Regression**	**Response: Presence of Metabolic syndrome**					Response: Presence of Metabolic syndrome				
**Predictor**	**OR** ^**b**^	**95% CI**			**P value**	**OR** ^**b**^	**95% CI**			**P value**
***F*_*1*_*-offspring group***										
**OMD**	0.84	0.41–1.69			0.620	**1.78**	**1.03–3.07**			**0.039**
**OFD**	1.09	0.57–2.07			0.792	0.85	0.46–1.58			**0.604**
**ONoPD**		Reference					Reference			
***Age*^*c*^*(per year increase)***	1.00	0.98–1.03			0.828	**1.03**	**1.01–1.06**			**0.006**
***BMI (per kgm*^*-2*^*increase)***	**1.18**	**1.09–1.29**			**<0.0001**	**1.21**	**1.13–1.30**			**<0.0001**
***Body-fat (per % increase)***	**1.12**	**1.05–1.19**			**0.001**	1.03	1.00–1.06			**0.063**
***SIMD quintile***										
**1 (Most deprived)**	1.84	0.79–4.25			0.155	1.66	0.82–3.36			**0.155**
**2**	1.43	0.67–3.06			0.356	**1.89**	**1.00–3.56**			**0.048**
**3**	1.35	0.65–2.80			0.420	1.38	0.70–2.74			**0.353**
**4**	1.57	0.85–2.92			0.152	1.02	0.54–1.93			**0.958**
**5 (Least deprived)**		Reference								
***Hips (per cm increase)***	Not included in model					1.01	0.98–1.05			**0.467**
***Smoking status***	Not included in model					0.81	0.60–1.09			**0.166**
***Sibling in study***	Not included in model					1.55	0.88–2.75			**0.131**

Univariate and logistic regression analysis of subjects in the F_1_-offspring group with metabolic syndrome (defined as 3 or more of the International Diabetes Federation criteria [18]).

Predictors with P values of <0.05 are highlighted in bold. All parameters included in the models are shown.

^a^ χ^2^ and P values from Chi-square analysis of OFD vs. ONoPD only.

^b^ OR are given as per unit change in the predictor

^**c**^ Age was used as a continuous variant in the model

We identified parameters associated with MetS in the F_1_-offspring, within each sex (Table 2). The final logistic regression model for males comprised: F_1_-offspring group, age, BMI, percent body-fat and SIMD; in females, the same parameters were used with hips, smoking status and sibling in study also included in the model. In males, predictors of MetS were: BMI (18% increased odds per kgm^-2^) and percent body-fat (12% increased odds per percentage increase in body-fat) but not SIMD or age. In females, predictors of MetS were: OMD (78% increased odds), BMI (21% increased odds per kgm^-2^) and age (3% increased odds per year). SIMD quintile 2 had an 89% increased odds in females but the confidence intervals were large and no other quintile had any effect.

### Effects of F_0_-parental diabetes on F_1_-offspring body and biochemical measurements

**Fig 2:**
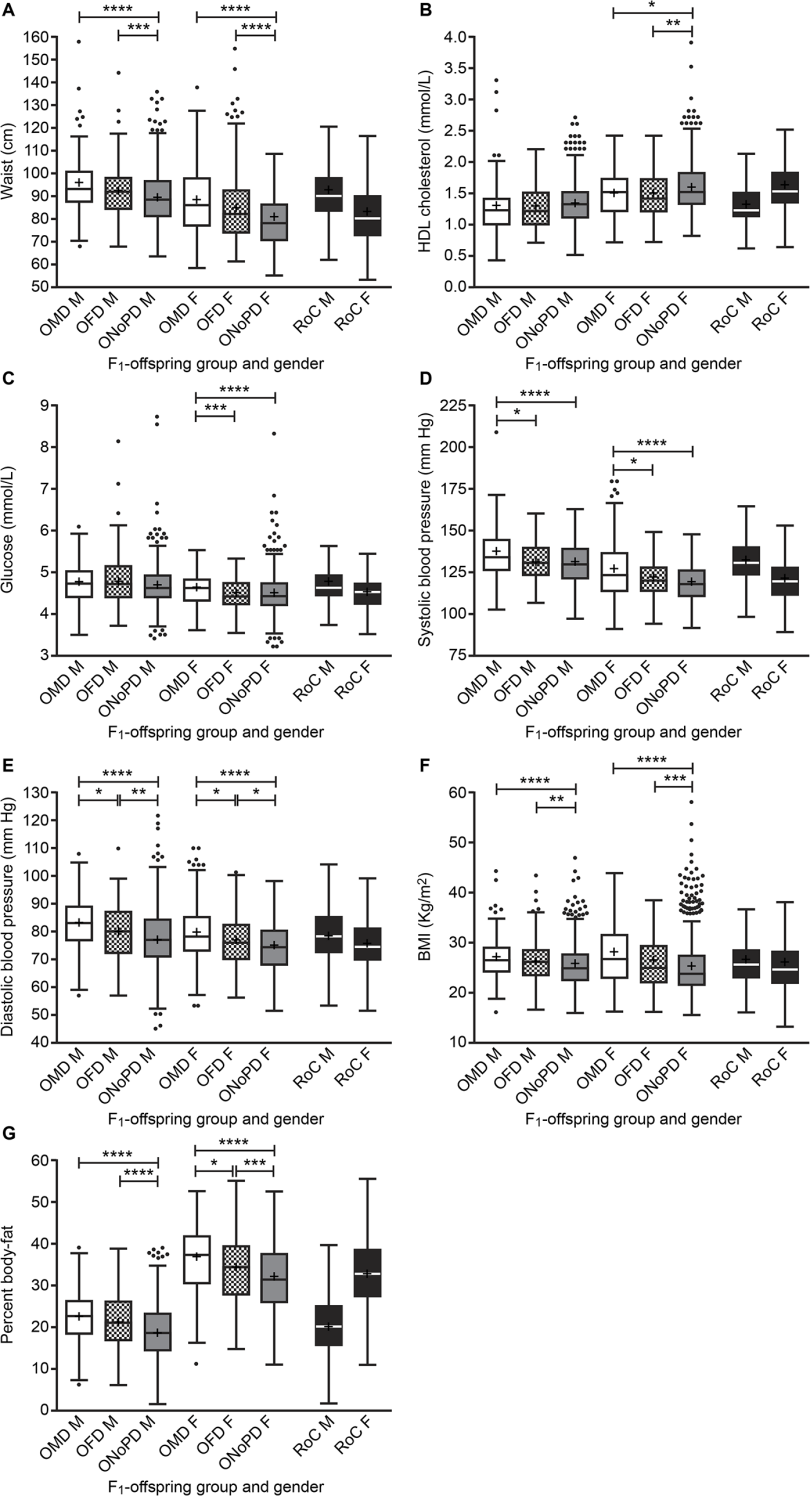
Body measurements, blood biochemistry and blood pressure measurements in offspring by parental group. Box-and-whiskers plots show median and IQR (‘**+’** within the box denotes mean value) for the OMD (white boxes), OFD (hatched boxes) and ONoPD (grey boxes) in males (left) and females (right); box-and whiskers for rest of cohort (RoC, i.e. all those without diabetes excluded from the F_1_-offspring groups, [black boxes] for males and females) are also shown for comparison. Graphs are shown for Waist (A), HDL-cholesterol (B), Glucose (C), Systolic-blood pressure (D), Diastolic-blood pressure (E), BMI (F) and Percent body-fat (G). Groups were compared by Kruskal-Wallis, with level of significance between the groups indicated (* P<0.05, **P<0.01, *** P<0.001, **** P<0.0001).

MetS is a cluster of factors that increase the risk of cardiovascular disease. We concentrated on the individual criteria of MetS and the factors influencing its development in univariate analyses of body measurements, blood biochemistry and blood-pressure between the F_1_-offspring groups, for each sex (Fig 2):

Waist measurements were significantly larger in male and female OMD and OFD compared with ONoPD (Kruskal-Wallis P<0.0001 for both sexes, Fig 2A).

HDL-cholesterol levels were significantly higher in female, but not male, ONoPD compared with OFD or OMD (Kruskal-Wallis P<0.0001, Fig 2B).

Glucose levels were significantly higher in female, but not male, OMD compared with OFD or ONoPD (Kruskal-Wallis P<0.0001, Fig 2C).

Systolic blood-pressure was significantly higher in female and male OMD compared with OFD or ONoPD (Kruskal-Wallis P<0.0001 for both sexes, Fig 2D).

Diastolic blood-pressure was significantly higher in female and male OMD compared with OFD or ONoPD; OFD had higher levels than ONoPD (Kruskal-Wallis P<0.0001 for both sexes, Fig 2E).

BMI was significantly lower in male and female ONoPD compared with OMD or OFD (Kruskal-Wallis P<0.0001 for both sexes, Fig 2F).

Percent body-fat was significantly higher in male and female OMD or OFD compared with ONoPD (Kruskal-Wallis P<0.0001 for both sexes); in females OMD also had higher levels than OFD (Fig 2G).

There were differences between F_1_-offspring groups for height, weight, hips, WHR, total cholesterol and THCR (data not shown). There were no significant differences between F_1_-offspring groups for sodium, potassium, urea or creatinine.

### Correlation between BMI of F_1_-offspring and F_0_-parental BMIs

We investigated whether there were any correlations between the offspring BMIs and maternal and paternal BMIs for each of the F_1_-offspring groups. BMIs of OMD significantly correlated with maternal BMIs in males (r = 0.2264, P = 0.0105) and females (r = 0.2202, P = 0.0023). However there was no correlation between BMIs of OMD and paternal BMIs in either males or females. BMIs of OFD significantly correlated with paternal BMI in males (r = 0.2356, P = 0.0032) and females (0.3838, P<0.0001). However, a significant correlation between BMIs of OFD and maternal BMIs was only seen in females (r = 0.4167, P<0.0001) and not males. BMIs of ONoPD significantly correlated with maternal BMIs in both males (r = 0.2271, P<0.0001) and females (r = 0.3268, P<0.0001) as well as with paternal BMIs in both males (r = 0.2306, P<0.0001) and females (r = 0.2465, P<0.0001).

### Intrauterine exposure to maternal diabetes

F_1_-offspring exposed *in utero* (EIU) to maternal diabetes (N = 18) were those whose mothers developed GDM (n = 10) or whose mothers had pre-existing T1DM (n = 8) during the index pregnancy. We compared the body and biochemical measurements between EIU and other OMD: the only difference seen between EIU and the other OMD was lower diastolic blood-pressure in male EIU (Kruskal-Wallis p = 0.0289, data not shown).

### Effect of having a grandparent with diabetes

We identified 127 F_2_-grandchildren of F_0_-grandparents with diabetes; all the F_0_-grandparents had T2DM. The median age for all the F_2_-grandchildren was 23 years (IQR 20–27). There was no difference in age for male F_2_-grandchildren, but in females GMGMD and GNoGPD were significantly older than GMGFD (Kruskal-Wallis, P = 0.0068). The demographic and social data showed no differences when compared between the F_2_-grandchildren groups by sex, apart from smoking habit, where a higher percentage of GNoGPD had never smoked and a lower percentage of GNoGPD were ex-smokers (Kruskal-Wallis P = 0.0001 for both sexes).

No differences in biochemical, body measurements or blood-pressures were seen when all those with a grandparent with diabetes were combined and compared with the GNoGPD group by sex, nor when grandchildren with maternal or paternal grandparents with diabetes were compared (data not shown). Differences were seen in male, but not female, grandchildren when compared by the defined grandparent groups:

Percent body-fat was significantly higher in male GPGFD (n = 5) than GPGMD (n = 19) and GNoGPD (n = 109, Kruskal-Wallis P = 0.0158);

Diastolic blood-pressure was significantly higher in male GPGMD than GMGFD (n = 22, Kruskal-Wallis P = 0.0318);

Total cholesterol levels were significantly higher in male GPGFD and GMGMD (n = 18) than in male GMGFD (Kruskal-Wallis P = 0.0005);

THCR were significantly higher in male GMGMD than GMGFD (Kruskal-Wallis P = 0.0033).

There were no differences in height, weight, WHR, HDL-cholesterol or systolic blood-pressure; differences in BMI, waist and hip measurements did not quite reach significance (Kruskal-Wallis, p = 0.0790, p = 0.0694 and p = 0.0530, respectively).

Nine grandchildren had MetS (5M, 4F) from GMGFD (n = 2, 5.4%), GMGMD (n = 2, 6.7%) and GNoGPD (n = 5, 2.4%) but differences were not significant. We were unable to do any further analysis of F_2_-grandchildren data due to the small numbers in each group.

## Discussion

The primary aim of this study was to investigate the long-term metabolic health effects of parental diabetes on adult offspring. In females, having a mother with diabetes increased the odds of MetS. Female OMD also had lower HDL-cholesterol and higher glucose levels than the other groups. In males, the odds of MetS were not raised by maternal diabetes. Having a father with diabetes did not increase the odds of MetS in either male or female offspring. However, both male and female offspring of either parent with diabetes had raised blood-pressure, larger waists, higher BMI and increased percent body-fat than those without a parent with diabetes.

This was a cross-sectional study of a Scottish family-cohort [15,16] across two generations, rather than a birth-cohort. The data-linkage of GS:SFHS subjects with their own medical records of diabetes ensured correct classification of the F_0_-generation diabetes-status: i.e. which parent/grandparent had diabetes and date of diagnosis. We used adult offspring and grandchildren who did not have diabetes themselves to reduce any potential confounding by diabetes disease-processes on metabolic health.

The F_1_-offspring groups were not well-matched in terms of age, as OMD and OFD were significantly older than ONoPD. This is likely to be due to the diagnosis of T2DM in older parents with higher BMIs and who would, therefore, also have older offspring. It is possible, and even quite likely, that some of the parents of ONoPD will be diagnosed with diabetes in the future. We had hoped to be able to compare any effects on the offspring of the different diabetes types, but the numbers of parents with T1DM were too small. We did see a temporal effect and the increase in T2DM diagnoses in Scotland over recent years [12] was very apparent in this study.

MetS was used to indicate the severity of the parental effect and likelihood of future cardiovascular events. Only 9% of all F_1_-offspring had MetS, a level similar to the 1958 Birth Cohort [19] where prevalence was 8–9% in subjects aged 45 years, 10 years older than the median age of the F_1_-offspring in this study. Due to the lack of triglyceride measurements this level of MetS is probably under-reported, as ~30% of OMD and OFD and ~20% of ONoPD met two (of four) MetS criteria: had triglyceride data been available, the occurrence of MetS would have been higher, highlighting the increased levels of MetS in this population.

Higher percentages of both male and female OMD and OFD had MetS than ONoPD, although after adjustment for confounders, the effects of paternal diabetes on all offspring and maternal diabetes on male offspring disappeared. These data suggest that maternal diabetes independently affects the odds of MetS, equivalent to that of an increase in BMI of ~3–4 kgm^-2^ in females, and that the sex of the offspring is important. MetS was also associated with the offspring’s own body measurements as BMI was a co-predictor in both sexes, while percent body-fat in males and age in females were additional predictors. The univariate analyses of the individual factors also showed differences between the OMD and other groups in females, especially for HDL-cholesterol and glucose, both criteria for MetS. Thus the sex dichotomy of MetS was borne out in its individual parameters. In males, there were fewer predictors for MetS and fewer differences in the individual parameters between groups. The particular combination of factors defining MetS may be more important than any one specific factor, in either sex. Others have also found sex-differences in the prevalence of MetS and its components in Caucasians [20], which may also reflect sex differences in developmental programming described in animal models and human epidemiological studies [21].

Maternal and paternal diabetes showed different cardiovascular effects in F_1_-offpsring as, compared with OFD, OMD had higher systolic and diastolic blood-pressures in both sexes. This was also apparent in (and possibly explains) the comorbidity data at recruitment into GS:SFHS, where a significantly higher proportion of OMD had already been diagnosed with heart disease, high blood pressure or stroke, and was seen in both sexes. Longer-term studies comparing children of mothers or fathers with diabetes showed that maternal effects were stronger [2], but that offspring of fathers with diabetes had higher BMI than offspring of healthy parents [3], in accordance with our results. Similarly, circulatory diseases have been found to be more prevalent in adult offspring of mothers with any diabetes, but also present in offspring of fathers with T2DM [4]. In a mouse-model of the effects of parental diabetes on pups without diabetes, metabolic parameters were affected by maternal diabetes and worsened with age, whereas skeletal development was affected by paternal diabetes and improved with age [22]. A study of fetal malformations in diabetic-rat pregnancy suggested that the maternal environment was not solely responsible for the effects of diabetes on offspring but that paternal genetics also contributed [23]. Thus our data and these studies suggest that both maternal and paternal diabetes affect adult offspring, but that maternal diabetes has stronger effects, although mechanisms are unclear.

While maternal diabetes during pregnancy leads to higher offspring birth-weight [5,7], offspring of fathers with diabetes have lower birth-weights [10,11], as do offspring of mothers who developed diabetes >10 years after childbirth, supporting the hypothesis that genetic factors contribute to the risk of decreased prenatal growth [11]. Our birth-weight data (for offspring born 1980–1992) showed no significant differences between offspring groups, but were not corrected for gestational age. Differences may also have been masked by the average birth-weight of babies in Scotland increasing over the same time-period and the fact that mothers with diabetes also had the shortest height and highest deprivation levels, both of which reduce offspring birth-weight [24].

The long-term health effects of parental diabetes on their offspring will be affected by shared lifestyle habits, diet, physical activity and stress levels. These data were not available but we would not expect to see specific lifestyle differences between OMD and OFD. The increase in parental T2DM diagnoses between 2000 and 2011 suggest that the pre-diagnosis dietary and exercise habits of these parents may have been well-established during the childhood and adult years of the offspring. The higher material deprivation of the OMD group might also contribute to differences in diet, levels of exercise and smoking [25]. We have no information on whether either parent remained with their offspring as they were growing up. It is possible that offspring of families where the mother had diabetes may have been more influenced by the mother’s diet-choices, especially if she prepared most of the family meals, than those where the father had diabetes. However, for offspring from areas of similar deprivation, we have assumed that contributions of shared lifestyle-factors would be similar in families with a mother with diabetes compared with families where the father had diabetes. The sex dichotomy within OMD is important and suggests that our results cannot be purely attributed to lifestyle similarities between parents and offspring.

We looked at parental BMI to see whether there was a relationship to that of their offspring. It was no surprise to find that maternal BMIs of OMD and paternal BMIs of OFD were higher than that of the other groups, as obesity is a key risk factor for diabetes. We did find that for the ONoPD, less than 30% of the parents had a normal BMI (i.e. of <25.0), which would suggest that a number of these parents are likely to be diagnosed with diabetes in future years. This was also true for the parent without diabetes in the OMD and OFD groups. Others have suggested that the BMI of both parents may influence the BMI of their offspring at different ages [26,27] and also the risk of later cardiovascular disease [28]. The BMIs of both parents correlated with the BMIs of the ONoPD group but the OMD BMIs only correlated with their maternal BMIs and not the paternal BMIs. Similarly the OFD BMIs correlated with their paternal BMIs but only their maternal BMIs in females and not males. These results suggest that genetic factors from the diabetes-affected parent, may have more of an influence on the offspring than that of the other parent. A recent study of BMI has shown a parent-of-origin-effect of SNPs in specific genes on the offspring, as the effects of these SNPs within maternal and paternal alleles were in opposite directions from each other [29]. Another potential mechanism is via imprinted genes, which are those which are expressed only from either a maternally- or paternally-transmitted copy, depending on the gene in question, with the inactive allele silenced by DNA methylation [30]. A recent study has shown that offspring of obese mothers and fathers have different methylation patterns on imprinted genes compared with offspring of lean parents [31]. Such mechanisms might affect long-term body composition and may also explain some of the sex-dichotomy seen in our results. Many imprinted genes involved in fetal growth are expressed in the placenta: paternally-derived imprinted genes promote fetal growth, whereas maternally-derived genes limit fetal growth to protect maternal resources [32]. Nutrition may affect imprinted genes: female offspring of male rats fed a high-fat diet had impaired glucose tolerance and higher insulin resistance [33], possibly through epigenetic changes in sperm at conception [34]. Offspring of rats fed a high-fat diet during pregnancy had an increased risk of obesity or metabolic syndrome, a trait which was transferred to two subsequent generations through the paternal lineage [35].

The number of EIU (n = 18) was smaller than expected: only 0.6% of those born 1980–1992. The prevalence of diabetes in pregnancy (both GDM and pre-existing diabetes) during the same time-period was 1% in North Dakota, USA [36]. Using this rate for the SMR02-linked data gives an expected number of EIU as 27. At the current prevalence of diabetes in women of reproductive age (5% [12]), the expected number of EIU in the SMR02-linked data would be ~135. Although diabetes-related pregnancy problems were recognized in the 1960s, routine glucose-tolerance testing in pregnancy is relatively recent. Since 2010, lower glucose-thresholds for GDM diagnosis have been adopted in Scotland [37]; thus GDM that would be identified under current criteria may have remained undiagnosed, explaining our lower rate of intrauterine exposure. Despite the smaller-than-expected numbers we did not find any overall increased effect of intrauterine diabetes exposure on the offspring.

We did find some evidence that grandparental diabetes may have an effect on their grandsons’ percent body-fat, total cholesterol and diastolic blood-pressure, but the numbers were too small to draw any robust conclusions. Others have shown a grandparental effect on metabolism: food-abundance in the pre-pubertal period in childhood of paternal grandfathers affected the risk of diabetes in their male grandchildren [38], while paternal grandmothers’ food-abundance affected their granddaughters, but not maternal grandparents [39,40]. Potential grandparental effects need to be investigated in much larger cohorts.

In conclusion, our study suggests that maternal diabetes increases the odds of occurrence of MetS in female offspring but has less effect in male offspring. However, having a father or mother with diabetes affects body and biochemical measures and blood-pressure in adult offspring of both sexes. These results have implications for offspring of parents with diabetes, who may need to take extra care with their diet and levels of exercise to combat a predisposition for higher BMI and body-fat. This may also be particularly important for daughters of mothers with diabetes as they grow older to prevent development of metabolic syndrome.
